# Is Desarda technique suitable to emergency inguinal hernia surgery? A systematic review and meta-analysis

**DOI:** 10.1016/j.amsu.2020.11.086

**Published:** 2020-12-02

**Authors:** Abdourahmane Ndong, Jacques Noel Tendeng, Adja Coumba Diallo, Mohamed Lamine Diao, Saer Diop, Diago Anta Dia, Philippe Manyacka Ma Nyemb, Ibrahima Konaté

**Affiliations:** Department of Surgery, Gaston Berger University, Saint-Louis, Senegal

**Keywords:** Hernia, Inguinal, Desarda, Incarceration, Strangulation, Surgery

## Abstract

**Background:**

Despite the fact that Lichtenstein is the gold standard for uncomplicated inguinal hernia, the use of mesh in an emergency context remains controversial. Pure tissue repairs have an essential role in the management of incarcerated or strangulated inguinal hernia. To date, there has been little agreement on what is the best surgical technique suitable for emergency hernia surgery. This systematic review aims to evaluate the efficacy and safety of the pure tissue Desarda technique for emergency inguinal hernia repair.

**Methods:**

A complete search of electronic databases including PubMed/Medline, Web of Science, Embase and, Cochrane library was realized. Newcastle-Ottawa-Scale (NOS) (selection and outcome criteria) was used for quality assessment of included studies. The pooled prevalence of post-operative complications (surgical site infection, hematoma/seroma, chronic pain and, recurrence rate) was estimated.

**Results:**

We included 5 studies from different countries. There were 2 randomized controlled trial and 3 observational cohort studies. Totally, there were 199 patients with a mean age of 57.6 years. Male patients were predominant (n = 196). The pooled prevalence of surgical site infection and hematoma/seroma was respectively 16.56% (95% CI: 11.74–22.39) and 12.43% (95%CI: 6.90–20.108). The pooled prevalence of chronic pain and recurrence was respectively 4.35% (95% CI: 1.04–11.47) and 2.10% (95%CI: 0.61–5.14).

**Conclusions:**

In summary, Desarda technique is feasible in emergency context with good results. We found any particularly important rate of complications considering the surgery in emergency context. Further studies should be realized to raise the level of evidence.

## Highlights

• Desarda technique is feasible in emergency context with good results.
• Any particularly important rate of complications was found considering the surgery in emergency context.
• Further studies with adequate number of patients and duration of follow-up should be realized to raise the level of evidence.

## 1 Introduction

One of the most significant subjects studied in abdominal wall surgery is inguinal hernia. Its management is very codified. Lichtenstein and laparo-endoscopic repair are the most recommended for uncomplicated primary inguinal hernia [1].

The main factors evaluating efficient hernia surgery are not only the rate of complications (recurrence and groin pain essentially) but also cost and time to return to normal activities [2]. Desarda technique is a non-mesh technique described first in 2001 [3]. This surgical technique uses a flap of external oblique aponeurosis in place of a mesh. Its singularity remains its low cost, no use of mesh, and less extensive dissection(3). Since its introduction, several studies compared Desarda to Lichtenstein technique which is considered as the gold standard for primary uncomplicated inguinal hernia. However, most of these studies, as shown in 3 different meta-analyses, have not found any difference between these two techniques in terms of short effectiveness for uncomplicated inguinal hernia [[4], [5], [6]].

Surgical technique for emergency inguinal hernia surgery remains an important area less documented. Emergency inguinal hernia can have different presentations. Incarceration is characterized by the irreducibility of contents and strangulation by the compromise of blood supply of the contents (e.g. bowel, omentum) [1,7]. The potential risk of necrosis and contamination of the surgical risk explains why the indications of prosthetic or pure tissue repair depend. In addition, this emergency context suggests less possibility for preparation and optimization for surgery. Since the use of mesh in an emergency context remains controversial, pure tissue repairs have an essential role in the management of incarcerated or strangulated inguinal hernia. To date, there has been little agreement on what is the best surgical technique or if the Desarda technique is suitable for emergency hernia surgery.

This systematic review aims to evaluate the efficacy and safety of the pure tissue Desarda technique for emergency inguinal hernia repair in terms of operative time, post-operative complications, length of hospital stay, chronic pain and, recurrence.

## 2 Methods

This systematic review with meta-analysis is realized to determine the place of Desarda technique in emergency hernia surgery by estimating the operative time, the prevalence of post-operative complication (surgical site infection, hematoma/seroma), length of hospital stay, chronic pain and, recurrence rate.

### 2.1 Search strategy

**Fig. 1:**
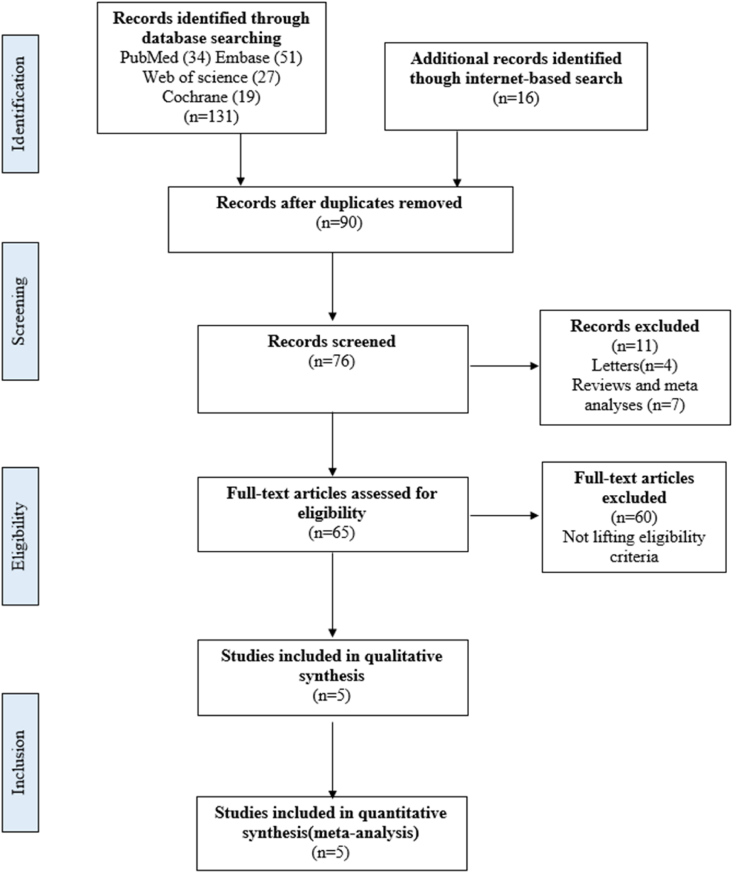
PRISMA flow diagram illustrating the search process and study selection.

A literature search for studies evaluating the Desarda technique of inguinal hernia surgery was conducted following the Preferred Reporting Items for Systematic Reviews and Meta-Analyses (PRISMA) guidelines as shown in Fig. 1 [8]. A complete search of electronic databases including PubMed/Medline, Web of Science, Embase and, Cochrane library was realized.

Keywords used in the search process included: “inguinal hernia,” “groin hernia,” “hernia,” “Desarda,” “Tissue-based,” “hernioplasty,” “emergency”, “incarcerated”, “strangulated”. The search was realized by using different combination of these terms

In addition, a manual search of relevant articles of the reference section of each publication was realized. Studies published between January 2001 and November 2020 were considered.

### 2.2 Study selection

We included studies with the following criteria (1)(): randomized trials or observational cohort studies (prospective or retrospective) (more than 5 patients) with a group of patients with strangulated or incarcerated inguinal hernia treated by Desarda Technique (2); Studies included at least one outcome of interest (operative time, post-operative complication (surgical site infection, hematoma or seroma), length of hospital stay, chronic pain and recurrence rate)(3); Studies in English or French language (4);Letters, reviews, conference abstracts, and duplicated studies were excluded.

### 2.3 Quality assessment

Newcastle-Ottawa-Scale (NOS) (selection and outcome criteria) was used for quality assessment of included studies [9]. A NOS score of 6 was considered as good quality, while 5 or less score as poor quality.

### 2.4 Data extraction

For each study, we extracted when available: type of study, number of patients evaluated, demographic characteristics of the patient population, and surgical outcome measures (operative time, surgical site infection, hematoma or seroma, length of hospital stay, chronic pain and recurrence rate).

### 2.5 Data analysis

Statistical analysis was done with Medcalc 14.8.1 software. A meta-analysis was performed to determine the pooled prevalence with the 95% confidence interval (CI) of complications (surgical site infection, seroma or hematoma, chronic pain and, recurrence) after emergency inguinal hernia surgery with the Desarda technique. Heterogeneity between studies was tested by I^2^ test. A random-effects model was used when I^2^ >50%; and a fixed-effects model when I^2^ ≤ 50%.

## 3 Results

### 3.1 Study characteristics

The PRISMA flow diagram illustrating the search process and study selection is represented at Fig. 1. We included 5 studies from different countries as detailed in Table 1. There were 2 randomized controlled trial and 3 observational cohort studies(10–14). The quality assessment of the different studies using the Newcastle-Ottawa Scale [9] is detailed in Table 2.

**Table 1:** Characteristics of the different included studies.

Study	Country	Study type	Quality of external oblique aponeurosis (EOA)	Number of patients	Mean age	Gender M/F	Follow-up (month)	Patients available for follow-up (%)
Hussain et al., 2017 [10]	Pakistan	Randomized Controlled Trial	Not Available (NA)	93	59.48 ± 14.76	93/0	1	NA
Pachauri et al., 2019 [11]	India	Observational cohort study	NA	30	52 ± 3	30/0	12	NA
Ansari et al., 2019 [12]	India	Randomized Controlled Trial	NA	41	NA	41/0	4	100
Sanna et al., 2020 [13]	Italy	Observational cohort study	Weak EOA excluded	15	68.8	12/3	6	100
Sagar et al., 2020 [14]	Bangladesh	Observational cohort study	Weak EOA excluded	20	50.25 ± 18.9	20/0	24	100

**Table 2:** Quality assessment of the different included studies according to the Newcastle-Ottawa Scale [9].

Study	Selection a	Outcome b	Score c
Hussain et al., 2017 [10]	***	*	4
Pachauri et al., 2019 [11]	***	*	4
Ansari et al., 2019 [12]	***	*	4
Sanna et al., 2020 [13]	***	**	5
Sagar et al., 2020 [14]	***	***	6

a The maximum score possible was 3 stars.

b Criteria used to assess outcome were at least 2 years' follow-up and a follow-up completion rate of at least 85%. The maximum score possible was 3 stars.

c The maximum score possible was 6 stars.

### 3.2 Patients characteristics

Totally, there were 199 patients with a mean age of 57.6 years. Male patients were predominant (n = 196). There were only 3 female patients.

In 2 studies, patients with weak external oblique aponeurosis (EOA) were excluded [13,14]. The duration of the follow-up was not the same in the different studies varying between 1 and 24 months. Only 3 studies realized a complete follow-up of all patients(12–14).

### 3.3 Post-operative outcomes

The repartition of operative outcomes among the different studies is represented in Table 3. The operative time was noted in 4 studies with an overall mean of 74.4 min [[10], [11], [12],^14^]. The length of hospital stay was reported in 4 studies with a mean of 4.06 days [11,13,14]. No death was reported.

**Table 3:** Meta-analysis of post-operative complications.

Complication	Number of studies	Total number of events	Total number of patients	Pooled prevalence	95%CI	I^2^	Model
Infection	5	32	199	16.56	11.74–22.39	0	Fixed
Hematoma or seroma	4	13	106	12.43	6.90–20.08	55.20	Random
Chronic pain	3	3	76	4.35	1.04–11.47	17.41	Fixed
Recurrence	5	3	199	2.10	0.61–5.14	0	Fixed

**Fig. 2:**
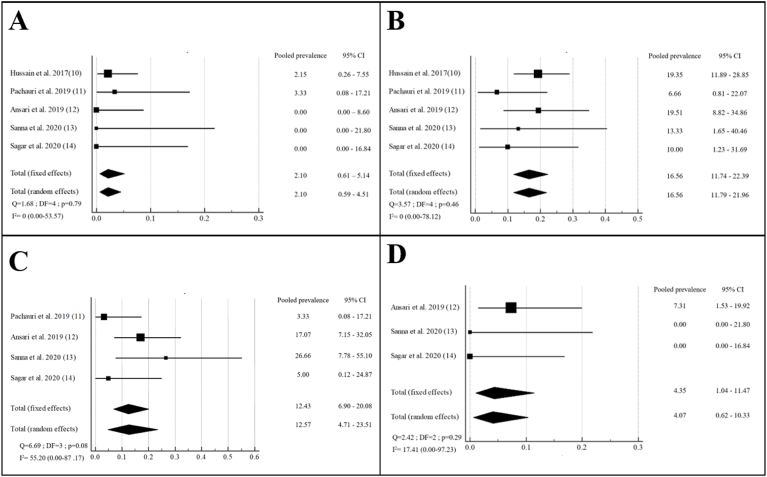
Meta-analysis for recurrence rate (A), surgical site infection (B), hematoma or seroma (C) and chronic pain (D).

The pooled prevalence of surgical site infection and hematoma/seroma was respectively 16.56% (95% CI: 11.74–22.39) and 12.43% (95%CI: 6.90–20.108). The pooled prevalence of chronic pain and recurrence was respectively 4.35% (95% CI: 1.04–11.47) and 2.10% (95%CI: 0.61–5.14). The forest plots detailing these pooled prevalence are represented at Fig. 2.

## 4 Discussion

Hernia is a major public health problem and an important global burden disease. Inguinal hernia is a large part of it with a worldwide prevalence of 8% [15]. It can be a life-threatening condition when strangulated with the risk of bowel necrosis, peritonitis and, sepsis. This explains why emergency inguinal hernia surgery is particular. The emergency presentation frequency varies but is more found in low- and middle-income countries. Some studies in resources-limited settings, even found that one-third of inguinal hernia were operated when incarcerated or strangulated [16].

The best surgical technique should be the one considering scientific evidence and cost-effectiveness [17]. Lichtenstein technique is considered widely as the gold standard for uncomplicated and complicated inguinal hernias. According to the WSES guidelines for emergency repair of complicated abdominal wall hernias, the mesh can be used for incarcerated inguinal hernia even if the surgical field is classified as clean-contaminated [7]. Nevertheless, questions have been raised about the safety of using a foreign body in an emergency context with the risk of infection. In addition, recent studies about the Lichtenstein technique have heightened the possibility of a higher associated rate of chronic pain[18]. Hence, tissue-based repairs, particularly Desarda technique, have always their place for emergency inguinal hernia surgery. This is particularly true in developing countries where it is estimated that more than 80% of inguinal hernia surgery are pure tissue repair (Modified Bassini, MacVay or Desarda) [15,19,20]. In that sense, Desarda technique is a great example because it is a tension and mesh-free repair [2]. Besides, 3 recent meta-analyses, have not found any difference between these two techniques (Desarda vs Lichtenstein) in terms of short effectiveness for uncomplicated inguinal hernia [[4], [5], [6]].

However, the recent International Guidelines for groin hernia management raised the urgent need of more research about emergency treatment of groin hernia [1]. In fact, there is a low level of evidence about this condition with the current state of literature. It is truer about the evaluation of Desarda technique for emergency surgery. We have found no controlled studies which comparing Desarda and Lichtenstein technique for emergency inguinal hernia repair. The 2 RCT included in our review were comparing Desarda to Darning and Bassini technique which are all tissue-based repair [10,12].

We have found a mean operative time of 74.4 min. Even if this duration is longer compared to elective surgery, Desarda repair is considered as a technique easily reproducible with a shorter learning curve [21].

The pooled prevalence of surgical site infection was 16.56% (95% CI: 11.74–22.39). This relatively high rate can be explained by the emergency context surgery [22]. Besides, most of the studies in our review (4 out 5) were realized in low and middle incomes countries(10–12,14).

This review estimated a rate of chronic pain at 4.35% (95% CI: 1.04–11.47). Nevertheless, only 3 studies evaluated it and with different assessment tools and duration of follow-up [[12], [13], [14]]. Further studies should focus more on its evaluation in emergency Desarda repair. Elective surgery with Desarda technique has already shown low rate absence of chronic pain(2,17).

Recurrence is an important component in the evaluation of the efficacy of a hernia surgical treatment and has been for a long time the only one considered criteria(21). Desarda technique is particularly associated with a low risk of recurrence even if it is a pure tissue repair. In fact, this technique strengthens the main anatomical element preventing hernia formation which is, according to the author, the aponeurotic extension in the posterior wall of the inguinal canal [23]. The pooled rate of recurrence was relatively low in our review at 2.10% (95%CI: 0.61–5.14) considering the emergency context. In fact, emergency admission is associated with a higher risk of recurrence even for mesh repairs [24,25]. However, most of the studies in our review did not have an adequate duration of follow-up.

In regards to these results, this technique has its advantages and should be more evaluated. In fact, there are a lot of barriers to use of recommended techniques (Lichtenstein, TAPPP, TEP) particularly in resources limited settings. The cost of mesh and endoscopic surgery materials can limit the use of techniques recommended by guidelines. In addition, the non-negligible rate of chronic pain when mesh is used, can lead to discuss the place of non-mesh repair such as Desarda technique.

Despite its numerous advantages, Desarda technique seems not to be generalizable in all types of patients and/or hernia [26]. It is not recommended in associated femoral hernia, complex hernias and, in the case of weak or thin external oblique aponeurosis. In our review, 2 studies excluded all patients with weak external oblique aponeurosis(13,14).

### 4.1 Strengths and limitations

This study is a systematic review evaluating Desarda technique for emergency inguinal hernia surgery. However, some limitations exist. In fact, the number of studies included in the systematic review was small (only 5) and there was no comparison with Lichtenstein technique which is considered by some as the gold standard. In addition, the follow-up was not long enough in the different studies to accurately estimate the recurrence rate. Also, assessment of chronic pain was not homogenous in the different studies.

## 5 Conclusion

Desarda technique has many advantages particularly in emergency context since it is considered as tension free repair without mesh. The results of this systematic review and meta-analysis suggest that the Desarda technique is feasible in emergency context with good results. Any particularly high rate of complications (considering the surgery in emergency context) was found. In addition, there is a lack of studies evaluating this technique for emergency inguinal hernia repair, despite the fact that it gave good result for uncomplicated hernias. The quality of the existing studies could be improved and further randomized controlled trials with adequate number of patients and duration of follow-up should be realized to raise the level of evidence.

## Ethical approval

This is review article, no need ethical approval.

## Sources of funding

The authors declare that this study had no funding resource.

## Author contribution

A Ndong, AC Diallo, JN Tendeng, M L Diao, S Diop and D A Dia conceived the study, collected, analysed data and drafted the manuscript. P M Ma Nyemb and I Konaté edited and reviewed the manuscript.

## Registration of research studies

We have registered our study with unique identifying number: **reviewregistry1035**.

## Guarantor

A Ndong is the guarantor.

## Consent

This is review article, no need ethical approval.

## Provenance and peer review

Not commissioned, externally peer reviewed.

## Declaration of competing interest

The authors declare that they have no conflicts of interests.
